# Utility of the optical quality analysis system for decision-making in cataract surgery

**DOI:** 10.1186/s12886-018-0904-1

**Published:** 2018-09-03

**Authors:** Jin Sun Hwang, Yoon Pyo Lee, Seok Hyun Bae, Ha Kyoung Kim, Kayoung Yi, Young Joo Shin

**Affiliations:** Department of Ophthalmology, Hallym University Medical Center, Hallym University College of Medicine, 948-1 Daerim1-dong, Youngdeungpo-gu, Seoul, 150-950 Korea

**Keywords:** Optical quality analysis system, Cataract, Objective scatter index, Modulation transfer function, Strehl ratio, Predicted visual acuity

## Abstract

**Background:**

A cataract is a common cause of vision impairment that requires surgery in older subjects. The Optical Quality Analysis System (OQAS, Visiometrics SL, Terrassa, Spain) assesses the optical quality of the eye in cataract patients. This study shows the role of the optical quality evaluation system for decision-making in cataract surgery. We investigated the clinical utility of the OQAS for decision-making in cataract surgery.

**Methods:**

Sixty-seven eyes from 67 patients undergoing cataract surgery and 109 eyes from 109 control subjects were compared. The best corrected visual acuity (BCVA) was measured. The objective scatter index (OSI), modulation transfer function (MTF), Strehl ratio, predicted visual acuity (PVA) 100%, PVA 20%, and PVA 10% were measured using the OQAS. The sensitivity and specificity of the different parameters were analyzed using the receiver operating characteristic (ROC) curve. The main parameters measured were sensitivity and specificity.

**Results:**

The BCVA, OSI, PVA 100%, PVA 20%, and PVA 10% were higher in the cataract group compared to those in the control group, while the MTF and Strehl ratios were lower (*p* <  0.001 for all). ROC analysis showed that the OSI had the largest area under the curve and that the sensitivity and specificity of the OSI were 83.9 and 84.6%, respectively, at the optimal cut-off point of 2.35.

**Conclusion:**

The MTF, OSI, Strehl ratio, PVA 100%, PVA 20% and PVA 10% may be useful parameters for preoperative decision-making in cataract surgery. The OSI appears to be the most effective parameter for this purpose.

## Background

Cataracts are a common cause of vision impairment in the older population, affecting the quality of vision and visual acuity and negatively impacting daily activities [1–3]. Although age-related cataracts progress slowly, cataract surgery is ultimately required [4]. The timing of surgery depends on weighing the benefits of surgery against the risks [5]. Advances in cataract surgery and intraocular lenses have improved surgical outcomes, promoted early visual rehabilitation, and reduced complications [4, 5]. These advances have led healthcare professionals to recommend cataract surgery to patients in early stages of the disease [4–6]. However, cataract surgery performed on eyes with good preoperative visual acuity has been linked to adverse visual results [5].

Visual functions, including visual acuity, glare and visual difficulties with daily activity, should be considered in the preoperative decision-making process for cataract surgery [7]. An accurate assessment of visual function facilitates the preoperative decision-making process for cataract surgery, resulting in a minimization of visual discomfort for patients. Indeed, the optical quality impairment caused by cataracts has become one of the major indications for cataract surgery [5].

The Lens Opacities Classification System III (LOCS III) is a nuclear opalescence grading system that is used to assess nuclear cataracts and has been shown to be a convenient and effective method in several studies [8–10]. However, this method is not able to provide information regarding optical quality or assess the visual quality impairment caused by cataracts [11]. The Optical Quality Analysis System (OQAS, Visiometrics, Terrassa, Spain) is based on the double-pass (DP) technique and was developed to evaluate vision quality objectively [12, 13]. The OQAS allows an objective assessment of intraocular scattering [14] and objectively measures the effect of optical aberrations and the loss of ocular transparency on the optical quality of the eye [15]. It provides optical quality parameters, such as the objective scatter index (OSI), modulation transfer function (MTF), Strehl ratio, and predicted visual acuity (PVA). However, a study of the utility of the OQAS for decision-making in cataract surgery has not been reported, and the most appropriate parameter for facilitating the decision-making process for cataract surgery has not been determined. Thus, in the present study, we investigated the usefulness of the OQAS for decision-making in cataract surgery.

## Methods

This study was a retrospective cross-sectional observational series. This study was approved by the Institutional Review Board (IRB) of Hallym University Kangnam Sacred Heart Hospital and adhered to the tenets of the Declaration of Helsinki for research involving human subjects. This study received a waiver of informed consent from the IRB of Hallym University Kangnam Sacred Heart Hospital because this study was a retrospective chart review study. The medical charts of patients who planned to undergo cataract surgery at Hallym University’s Kangnam Sacred Heart Hospital between October 1, 2014 and August 31, 2015 and those of the control subjects were retrospectively reviewed. Data from all patients were collected and analyzed. The cataract group included the patients who needed cataract surgery and planned to undergo the procedure because they had a decrease in vision due to the cataracts. They wished to undergo the cataract surgery, had a visual acuity less than 20/30, had any type of cataract greater than grade 2, or had a cataract that affected the patient’s lifestyle. They had normal retinal and corneal findings. The patients in the cataract group discussed the risks and benefits of cataract surgery with the surgeon, and then they decided to undergo the cataract surgery. During the same period, a gender-matched control group was included. The control group consisted of patients who visited the clinic for routine eye examinations, had minimal opacities in the lens, and normal retinal and corneal findings. Patients who had undergone additional intraocular procedures and patients with corneal abnormalities were excluded.

The best corrected visual acuity (BCVA) was assessed using the Hans visual acuity chart and refractive error with an auto kerato-refractometer (KR-8100, Topcon, Tokyo). Cataracts were classified using the LOCS III [8]. For this analysis, each subject was allocated an LOCS III grade based on the single highest score reported in each of the following categories: nuclear cataract (NC; on a scale from I to VI), cortical cataract (CC; on a scale from I to V), and posterior subcapsular cataract (PSC; on a scale from I to V) [8–10]. Mixed cataract was defined as a combination of any two types of opacity [9, 10].

Using an artificial pupil of 4.0 mm in diameter under mesopic conditions, the optical quality of the eyes was measured using the OQAS, which is an instrument based on the DP technique. The subject was asked to put his or her chin on the chinrest and fix the center of a figure. The examiner aligned the optical axis of the instrument with the subject’s pupil center. During the measurements, spherical errors were corrected by an incorporated optometer in the DP system, while external lenses were used to correct cylindrical errors ≥ − 0.50D [12]. The MTF, OSI, Strehl ratio, PVA 100%, PVA 20%, and PVA 10% were all measured using the OQAS. The MTF curve displays the percentage reduction of retinal image contrast at a variety of resolutions. The OSI quantifies the degree of ocular scattering caused by the loss of transparency in ocular structures, such as corneal haze, cataract, and vitreous opacities [16]. The acuity calculated using the OQAS represented optical characteristics of the eye, including aberrations and ocular scatter [16]. The maximum visual acuity was predicted for objects with 100, 20, and 10% contrast [16].

### Statistics

A two-sample t-test was used to compare the patients undergoing cataract surgery to the control subjects. The similarities and differences between cataract classification groups were determined using the Kruskal-Wallis test following the Mann-Whitney U test. Statistical significance was based on two-tailed statistical analyses, and probability values < 0.05 were considered statistically significant. Visual acuity was measured in terms of the logarithm of the minimum angle of resolution (logMAR). Receiver operating characteristic (ROC) analysis was used to calculate test sensitivity and specificity using SPSS 23.0 for Windows (IBM Corp., Chicago, IL). Comparison of the ROC curves was performed using the DeLong method from MedCalc version 11.4.4 statistical software (MedCalc Software, Mariakerke, Belgium).

## Results

Data were analyzed from 29 men and 38 women in the cataract group and 33 men and 76 women in the control group (*p* = 0.079, chi-square test). The mean age was 67.34 ± 8.18 years in the cataract group and 59.45 ± 10.53 years in the control group (*p* <  0.001, t-test). The mean logMAR visual acuity was 0.48 ± 0.41 in the cataract group and 0.08 ± 0.23 in the control group (*p* <  0.001, t-test). The lens opacity grade using the LOCS III was 0.31 ± 0.47 for NC, 0.19 ± 0.44 for CC, and 0.00 ± 0.00 for PSC in the control group and 1.75 ± 1.03 for NC, 1.79 ± 1.30 for CC and 0.68 ± 1.17 for PSC in the cataract group (*p* <  0.001 between cataract group and control group for all, t-test).

**Fig. 1:**
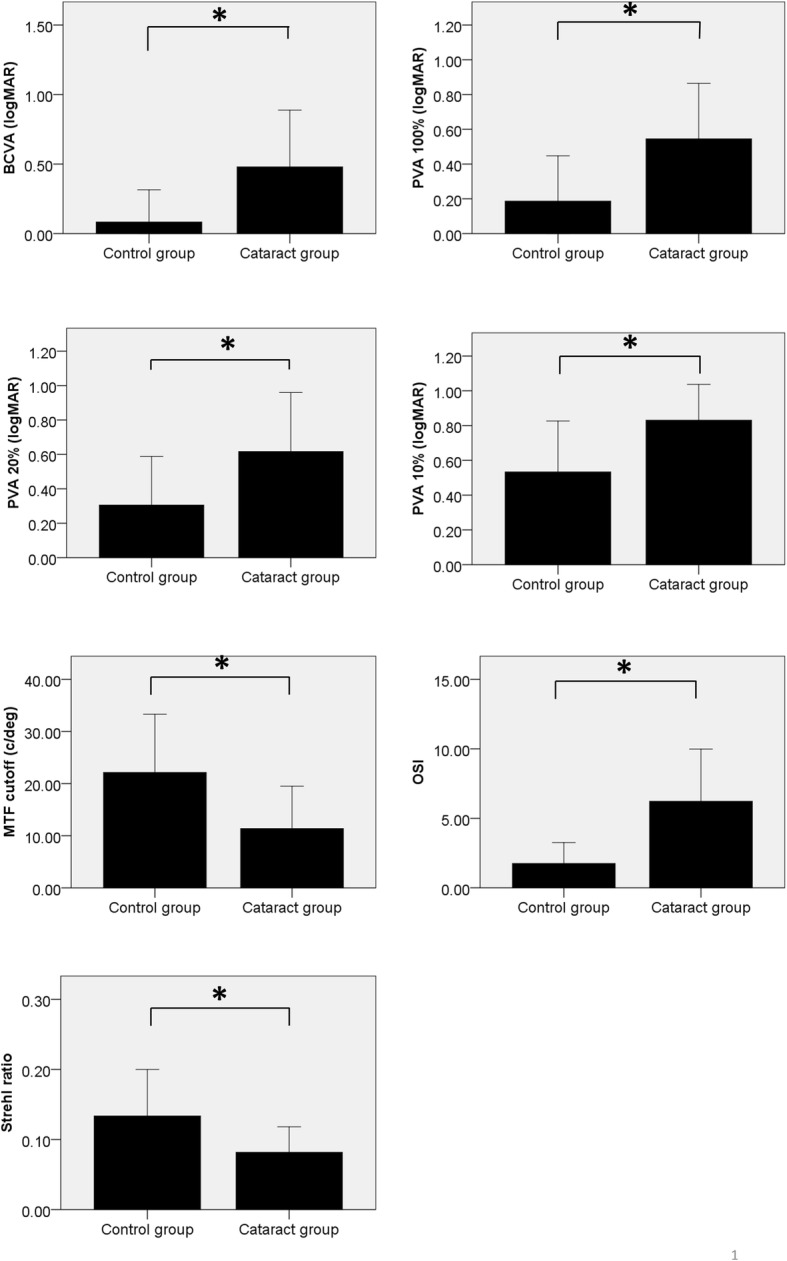
The Optical Quality Analysis System (OQAS) parameters in cataract and control groups are shown. Best corrected visual acuity (BCVA), predicted visual acuity (PVA) 100%, PVA 20%, PVA 10%, and objective scatter index (OSI) were higher in the cataract group than in the control group (*p* <  0.001 for all, t-test). The modulation transfer function (MTF) and Strehl ratio were lower in the cataract group compared to that in the control group (*p* <  0.001 for all, Student’s t-test). * statistically significant using Student’s t-test

Table 1 shows clinical findings for the subjects, including age, gender, and symptoms. There was no difference in gender between the control and cataract groups (*p* = 0.104, chi-square test). The control group was comprised of 109 eyes, while the cataract group was comprised of 67 eyes (from 67 patients). The BCVA (logMAR) was worse in the cataract group (0.48 ± 0.41) compared to that in the control group (0.08 ± 0.23; *p* <  0.001). Measurements obtained from the OQAS were compared between the cataract group and the control group (Fig. 1). The MTF was lower in the cataract group (11.38 ± 8.13) compared to that in the control group (22.14 ± 11.17; *p* <  0.001). The OSI was higher in the cataract group (6.23 ± 3.75) compared to that in the control group (1.75 ± 1.51; *p* <  0.001). The Strehl ratio was lower in the cataract group (0.08 ± 0.04) compared to that in the control group (0.13 ± 0.07; *p* <  0.001). The PVA 100% (logMAR) was higher in the cataract group (0.55 ± 0.32) compared to that in the control group (0.19 ± 0.26; *p* <  0.001). The PVA 20% (logMAR) was also higher in the cataract group (0.62 ± 0.34) compared to that the control group (0.31 ± 0.28; *p* <  0.001). Finally, the PVA 10% (logMAR) was higher in the cataract group (0.83 ± 0.21) compared to that in the control group (0.53 ± 0.29; *p* <  0.001).

**Table 1:** Comparison between control and cataract groups

	Control group	Cataract group						
		Total	*p*-value	CC	NC	PSC	Mixed	*p*-value
N (eyes)	109	67		18	20	9	20	
Age (year)	59.45 ± 10.53	67.34 ± 8.18	< 0.001*	63.39 ± 6.75	66.35 ± 11.34	69.67 ± 8.53	68.15 ± 5.23	< 0.001*
Male: female	33:76	29:38	†0.079	8:10	7:13	4:5	10:10	†0.408
Eye laterality								
Right: left	54:55	34:33	0.877	10:8	12:8	1:8	11:9	0.185
SE (D)	−0.01 ± 1.71	− 0.25 ± 3.28	0.522	0.87 ± 1.52	−1.00 ± 3.00	− 1.514 ± 7.36	0.05 ± 0.92	0.062
BCVA (logMAR)	0.08 ± 0.23	0.48 ± 0.41	< 0.001*	0.43 ± 0.46	0.54 ± 0.43	0.68 ± 0.61	0.44 ± 0.47	< 0.001*
MTF	22.14 ± 11.17	11.38 ± 8.13	< 0.001*	15.17 ± 8.94	9.68 ± 5.39	8.48 ± 6.03	10.98 ± 9.65	< 0.001*
OSI	1.75 ± 1.51	6.23 ± 3.75	< 0.001*	2.99 ± 1.35	7.58 ± 3.38	8.82 ± 4.10	6.61 ± 3.70	< 0.001*
Strehl ratio	0.13 ± 0.07	0.08 ± 0.04	< 0.001*	0.09 ± 0.03	0.08 ± 0.03	0.07 ± 0.027	0.08 ± 0.05	< 0.001*
PVA 100% (logMAR)	0.19 ± 0.26	0.55 ± 0.323	< 0.001*	0.36 ± 0.24	0.61 ± 0.30	0.67 ± 0.31	0.59 ± 0.35	< 0.001*
PVA 20% (logMAR)	0.31 ± 0.28	0.62 ± 0.34	< 0.001*	0.48 ± 0.29	0.72 ± 0.26	0.64 ± 0.42	0.62 ± 0.40	< 0.001*
PVA 10% (logMAR)	0.53 ± 0.29	0.83 ± 0.21	< 0.001*	0.78 ± 0.20	0.86 ± 0.18	0.90 ± 0.17	0.85 ± 0.24	< 0.001*

*NC* nuclear cataract, *CC* cortical cataract, *PSC* posterior subcapsular cataract, *SE* spherical equivalent, *BCVA* best corrected visual acuity, *MTF* modulation transfer function, *OSI* objective scatter index, *PVA* predicted visual acuity

*statistically significant using Student’s t-test, † the Pearson chi-square test

**Fig. 2:**
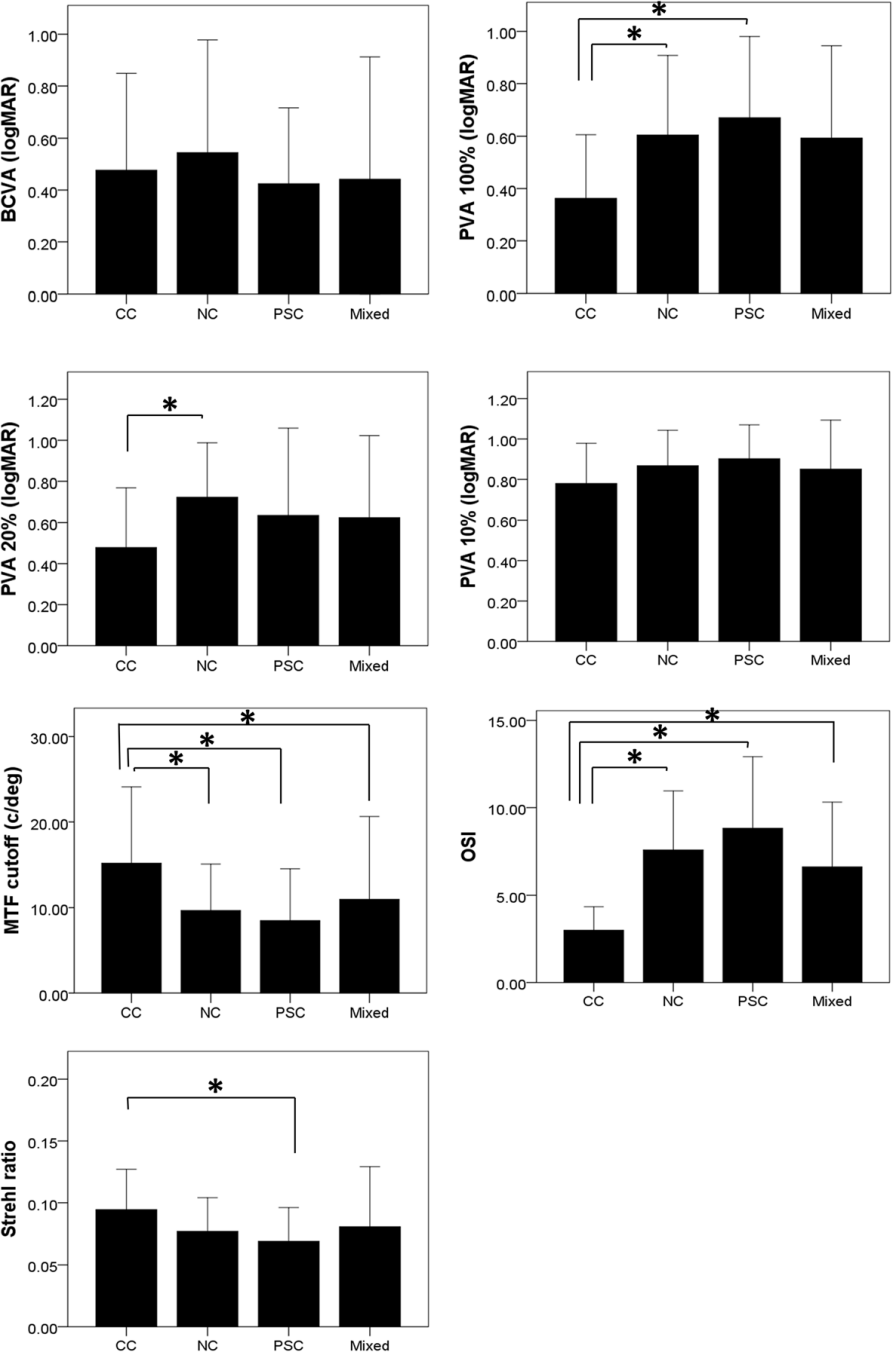
The Optical Quality Analysis System (OQAS) parameters grouped according to cataract classification are shown. The Kruskal-Wallis analysis of the data grouped according to cataract type revealed significant differences in best corrected visual acuity (BCVA), modulation transfer function (MTF), objective scatter index (OSI), Strehl ratio, predicted visual acuity (PVA) 100%, PVA 20%, and PVA 10% among the cataract types (*p* <  0.001 for all). The MTF was lower in the NC, PSC and Mixed groups compared to that in the CC group (*p* = 0.041, 0.035 and 0.048, respectively, Mann-Whitney U test). The OSI was higher in the NC, PSC and Mixed groups compared to that in the CC group (*p* <  0.001, 0.001, and 0.001, respectively). The Strehl ratio was lower in the PSC group compared to that in the CC group (*p* = 0.046). The PVA 100% was higher in the NC and PSC groups compared to that in the CC group (*p* = 0.048 and 0.035, respectively). The PVA 20% was higher in the NC group compared to that in the CC group (*p* = 0.022). No difference in the BCVA and PVA 10% was observed between cataract types. * statistically significant using the Mann-Whitney U test

Cataracts were classified into 4 types (Table 1). As a percentage of total cataracts studied, 29.9% were NC, 26.9% were CC, 13.4% were PSC, and 29.9% were mixed cataracts. There was no difference in gender between subgroups (*p* = 0.394, chi-square test). The Kruskal-Wallis analysis of the data grouped according to the cataract type revealed no significant differences in the BCVA, MTF, Strehl ratio, PVA 100%, PVA 20%, and PVA 10% between cataract types, whereas there was a significant difference in the OSI according to cataract type (*p* <  0.001; Fig. 2). The MTF was lower in the NC, PSC and Mixed groups compared to that in the CC group (*p* = 0.041, 0.035 and 0.048, respectively, Mann-Whitney U test). The OSI was higher in the NC, PSC and Mixed groups, compared to that in the CC group (*p* <  0.001, 0.001, and 0.001, respectively). The Strehl ratio was lower in the PSC group compared to that in the CC group (*p* = 0.046). The PVA 100% was higher in the NC and PSC groups compared to that in the CC group (*p* = 0.048 and 0.035, respectively). The PVA 20% was higher in the NC group compared to that in the CC group (*p* = 0.022). No difference in the BCVA and PVA 10% was observed between cataract types.

**Fig. 3:**
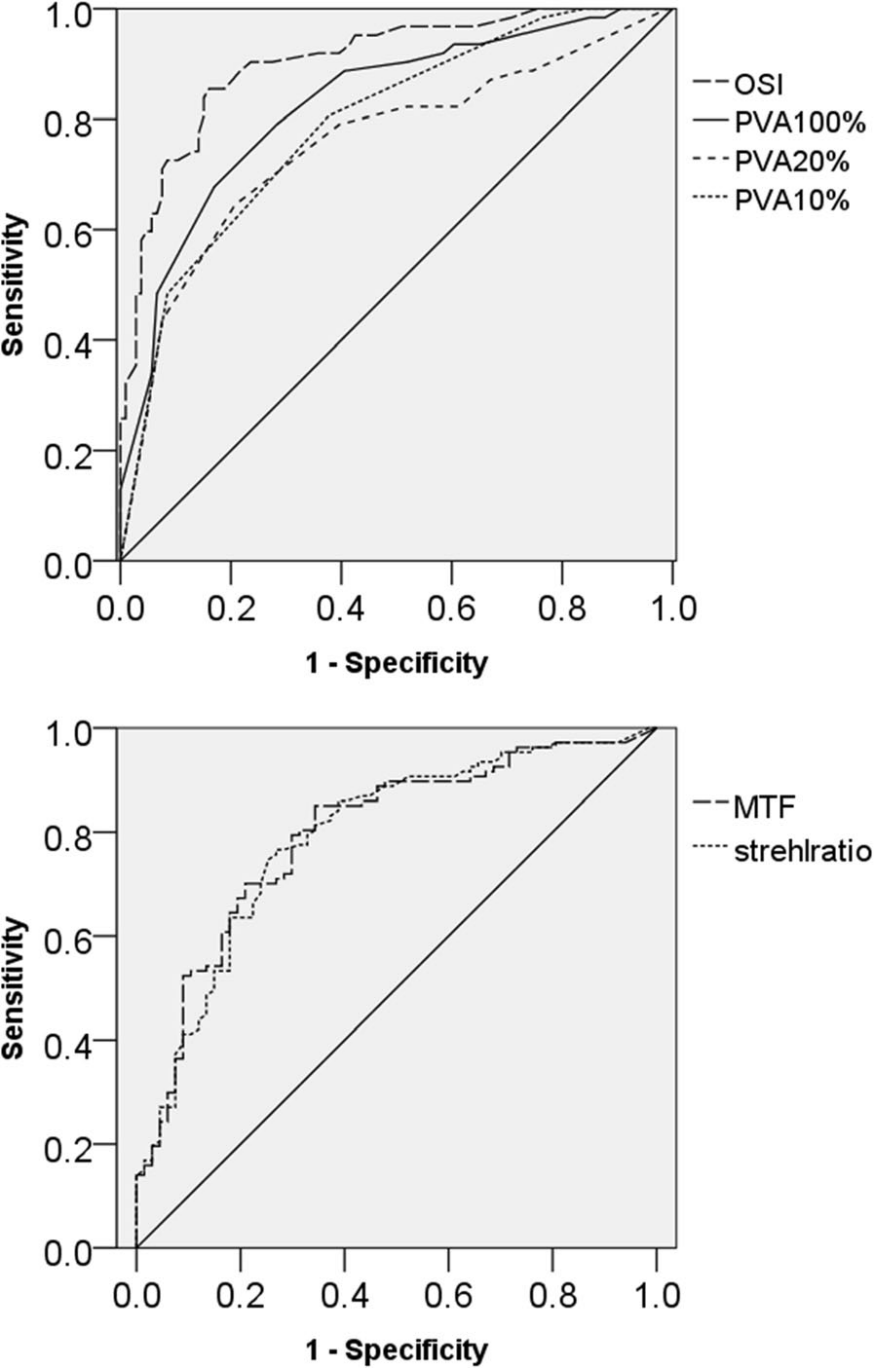
The Receiver Operating Characteristic (ROC) curves for the objective scatter index (OSI), predicted visual acuity (PVA) 100%, PVA 20%, PVA 10%, modulation transfer function (MTF) and Strehl ratio

According to the ROC curve analysis (Fig. 3), the area under the curve (AUC) was 0.900 (0.847–0.953) for the BCVA, 0.805 (0.733–0.876) for the MTF, 0.902 (0.853–0.951) for the OSI, 0.800 (0.727–0.873) for the Strehl ratio, 0.828 (0.761–0.896) for the PVA 100%, 0.749 (0.6669–0.833) for the PVA 20%, and 0.791 (0.720–0.862) for the PVA 10%. Overall, the OSI had the largest AUC. The AUC for the OSI was larger compared to that for the MTF, Strehl ratio, PVA 20% and PVA 10% (*p* <  0.001 for all, DeLong’s method). The sensitivity and specificity of the OQAS parameters for facilitating preoperative decision-making in cataract surgery are shown in Table 2. The sensitivity and specificity of the OSI at the optimal cut-off point of 2.35 were 83.9 and 84.61%, respectively.

**Table 2:** Receiver operating characteristic curves for the OSI, PVA100%, PVA20%, PVA10%, MTF and Strehl ratio

	Cut-off point	Sensitivity	Specificity	J-index	AUC (95% CI)	*p*-value
BCVA (logMAR)	0.2600	70.6%	95.2%	0.650	0.900 (0.847–0.953)	< 0.001*
MTF	16.15	79.3%	72.1%	0.514	0.805 (0.733–0.876)	< 0.001*
OSI value	2.35	83.9%	84.6%	0.685	0.902 (0.853–0.951)	< 0.001*
Strehl ratio	0.0955	71.0%	78.8%	0.498	0.800 (0.727–0.873)	< 0.001*
PVA100% (logMAR)	0.2600	79.0%	71.7%	0.507	0.828 (0.761–0.896)	< 0.001*
PVA20% (logMAR)	0.4600	64.5%	79.2%	0.437	0.749 (0.666–0.833)	< 0.001*
PVA10% (logMAR)	0.6600	80.6%	62.3%	0.429	0.791 (0.720–0.862)	< 0.001*

*AUC* area under the curve, *95% CI* 95% confidence interval, *BCVA* best corrected visual acuity, *MTF* modulation transfer function, *OSI* objective scatter index, *PVA* predicted visual acuity

*statistically significant

## Discussion

Prior to cataract surgery, an assessment of the patient’s discomfort resulting from the cataract and an objective evaluation of the consequent visual impairment are essential [5]. Optical quality has become an important factor for consideration during decision-making in cataract surgery because it has an effect on the quality of life [4]. The OQAS has been shown to provide robust and fully objective measurements of optical quality; i.e., not depending on subjective decisions [11]. Furthermore, the OQAS has previously been suggested to be helpful when used in combination with standard methods to improve cataract surgery scheduling [11]. This study investigated the usefulness of different optical quality measurements obtained using the OQAS in the preoperative decision-making process for cataract surgery.

In this study, measurements obtained from the OQAS were compared between the cataract group and a control group. The BCVA, PVA 100%, PVA 20%, PVA 10%, and OSI were higher in the cataract group than in the control group (*p* <  0.001 for all, t-test). In contrast, the MTF and Strehl ratio were lower in the cataract group compared to that in the control group (*p* <  0.001 for all, Student’s t-test). It is important to note that these parameters were also different among cataract types. According to previous reports, the MTF cut-off frequency and the Strehl ratio decreased, while the OSI increased with aging [17]. The optical quality of a patient’s eyes has been shown to degrade with cataract grade [18]. The MTF was determined from an iTrace and a DP system from the OQAS and differed significantly in a comparison between subjects with early cataract development and normal controls [19]. However, the correlations of the MTF with visual performance were higher for the OQAS system. Thus, the MTF derived from the OQAS has been suggested to be useful as an indicator of visual performance in eyes with cataracts [19]. The OSI, another parameter obtained using the DP system, has been shown to be correlated with the Scheimpflug-measured lens density, subjective lens grading, and cumulative dissipated energy. The measurement of the OSI may improve the preoperative evaluation of nuclear cataracts and help predict phacodynamics in cataract surgery [20].

Using ROC curve analysis, this study found that the AUC was the largest for the OSI. It has previously been reported that several objective measurements obtained using the OQAS, including the MTF cut-off, OSI and Strehl ratio, differ between eyes with cataracts and control eyes [11, 16, 20, 21]. The MTF curve, computed from the point spread function (PSF), displays the percentage reduction of the contrast of the retinal image at various spatial resolutions and represents the combined effects of high-degree optical aberrations and scatter [16]. It is the ability of a lens or ocular structure to transfer the object contrast to the image. It has been suggested that the MTF plots are associated with the subsystems that make up a complete electro-optical or photographic system [22]. The MTF is associated with tear film stability [23] or the type of intraocular lens [24]. The OSI is defined as the ratio between the integrated light intensity in the periphery and that around the central peak of the double-pass image [11]. The OSI reflects the degree of scattering caused by the loss of transparency in the cornea or lens [16]. The OSI gradation relates directly to the extent of visual degradation (forward scatter) [11]. The higher OSI value represents a higher level of intraocular scattering [16]. The OSI has been found to correlate with NC and PSC severity [16, 25]. The Strehl ratio is defined as the ratio of the peak intensity of a measured PSF to that of a perfect optical system [26, 27]. The Strehl ratio expresses the ability of the eye to form a point image on the retina when a point object is seen [28]. It is related to wavefront errors [29], aging [17] and characteristics of the intraocular lens [24].

In this study, the MTF was lower in the NC, PSC and Mixed groups compared to that in the control, and the OSI was higher in the NC, PSC and Mixed groups compared to that in the control and CC groups. Optical quality by OQAS was measured in a pupil diameter of 4 mm [30]. The NC and PSC are located at the center of the lens and disturb visual quality [31]. Thus, central opacity of the lens may have a greater effect on optical quality [30]. A CC may have less effect on optical quality of the lens because a CC affects the periphery of the lens [8, 32]. The PVA 100% and PVA 20% were higher in the NC, PSC and Mixed groups compared to that in the control group. An NC has opacities in the center, which may have an impact on the PVA 100% and PVA 20%.

Although the LOCS III grading system is still an economical and effective way to evaluate the severity of lens opacities, the OSI can be useful to assess the impact of cataracts on a patient’s vision objectively when there is a difference between patient symptoms and ocular examination findings [21]. It is suggested that OSI scores ≥3.0 can be helpful as a possible cut-off for preoperative decision making [21]. In contrast to this previous study, we employed an ROC analysis to determine the cut-off value for the decision-making process in cataract surgery. The use of ROC analysis to evaluate diagnostic tests is widespread [33].

According to the ROC curve analysis, the OSI had the largest AUC. The AUC for the OSI was larger compared to that of the MTF, Strehl ratio, PVA 20%, and PVA 10% (*p* <  0.001 for all, DeLong’s method). The AUC is an effective tool to assess sensitivity and specificity of diagnostic tests. The AUC summarizes the entire location of the ROC curve rather than depending on a specific operating point [34]. Thus, the OSI is the most accurate test for decision-making in cataract surgery. The Youden index (J-index) is used to determine the optimal cut-off point [35]. In our study, the sensitivity and specificity of the OSI were 83.9 and 84.6%, respectively, at the optimal cut-off point of 2.35. These results provided the rationale that cataract surgery may be postponed in eyes with low OSI scores, whereas cataract surgery is necessary in the eyes with high OSI scores. Visual functions should be considered in the preoperative decision-making process for cataract surgery [7]. The OQAS parameters directly relate with the visual degradation in any type of cataract [11, 13]. Because the cataract observed in a slit lamp examination is not always predictive of the actual visual impact, the OQAS parameters have the advantage of being able to predict the quality of the patient’s vision and provide it to the operator.

One limitation of this study is that the quality of life was not measured even though a discussion occurred between the patients and the doctor in determining the cataract surgery. Further study is necessary to evaluate the quality of life in the decision-making process for cataract surgery. Another limitation was that the control group was younger compared to the age of the cataract group. The change in the OQAS due to aging is mainly associated with a decrease in optical quality secondary to cataract formation or lens changes [36]; cataracts increase with aging [37]. Therefore, the age of the cataract group was higher in the normal group. Another source of optical quality changes due to aging is corneal changes. The high order aberration of the cornea due to aging is increased [38], which can be reflected in the change in optical quality [39]; however, it does not have much effect. In this study, the cornea could not have affected the optical quality because all subjects had normal corneal findings.

In this study, the cataract group consisted of the patients with cataracts requiring cataract surgery. Although the difference in the OQAS between nonsurgical cataracts and cataracts were previously evaluated [40], further study including the patients with early cataract development is needed to increase the clinical significance of the decision-making process.

## Conclusion

The MTF, OSI, Strehl ratio, PVA 100%, PVA 20% and PVA 10%, measured by the OQAS may be useful for preoperative decision-making in cataract surgery. Among these, the OSI is the most effective parameter for use in the decision-making process for the determination of the suitability of cataract surgery.

## Abbreviations

BCVA: best corrected visual acuity
CC: cortical cataract
DP: double-pass
LOCS III: Lens Opacities Classification System III
logMAR: logarithm of the minimum angle of resolution
MTF: modulation transfer function
NC: nuclear cataract
OQAS: Optical Quality Analysis System
OSI: objective scatter index
PSC: posterior subcapsular cataract
PVA: predicted visual acuity
ROC: receiver operating characteristic
